# A_3_ adenosine receptor regulates depression-like behaviors through astrocyte-nourished excitatory synapse formation

**DOI:** 10.1016/j.isci.2026.116843

**Published:** 2026-07-16

**Authors:** Bai Li, Meng Zeng, Jiahui Liu, Wanqing Huang, Dan Pan, Chunjie Xiao, Jing Du

**Affiliations:** 1 School of Medicine, Yunnan University, Kunming, Yunnan 650091, China; 2 The National Clinical Research Center for Mental Disorders and Beijing Key Laboratory of Mental Disorders, Beijing Anding Hospital, Capital Medical University, Beijing 100088, China

**Keywords:** major depressive disorder, A_3_ adenosine receptor, neuroligin-2, astrocyte, Synaptogensis

## Abstract

Abnormal excitatory synaptic function plays a critical role in major depressive disorder. In this study, we investigated the role of the A_3_ adenosine receptor (A_3_AR), previously implicated in affective disorders, in excitatory synapse formation within prefrontal cortical circuits. Using a corticosterone-induced mouse model of depression, we found that the adenosine receptor activator 3′-deoxyadenosine (3′dA) produced antidepressant-like effects and selectively increased A_3_AR expression, whereas fluoxetine did not. 3′dA also restored the synaptic organizers neuroligin-2 (NLGN2) and neurexin-2 and elevated BDNF, excitatory synaptic markers (synapsin-1, PSD-95, and GluR1), and dendritic spine density in the PFC. Notably, enhanced NLGN2 immunoreactivity co-localized with astrocytes exclusively in 3′dA-treated mice but not in fluoxetine-treated mice. Pharmacological blockade of A_3_AR with MRS-1191 prevented the 3′dA-induced behavioral and molecular changes in this corticosterone-induced depressive-like mouse model. Together, our findings identify A_3_AR as a key regulator of NLGN2 expression and excitatory synapse formation and suggest a potential target for antidepressant therapies.

## Highlights

• 3′-Deoxyadenosine (3′dA) produces antidepressant-like effects in a mouse model of depression
• 3′dA treatment is associated with increased astrocytic A_3_AR and NLGN2 expression in the PFC
• A_3_AR activation is associated with increased synaptic protein and spine density in PFC
• Pharmacological blockade of A_3_AR abolishes the antidepressant-like effects of 3′dA

## Graphical abstract

**Figure:**
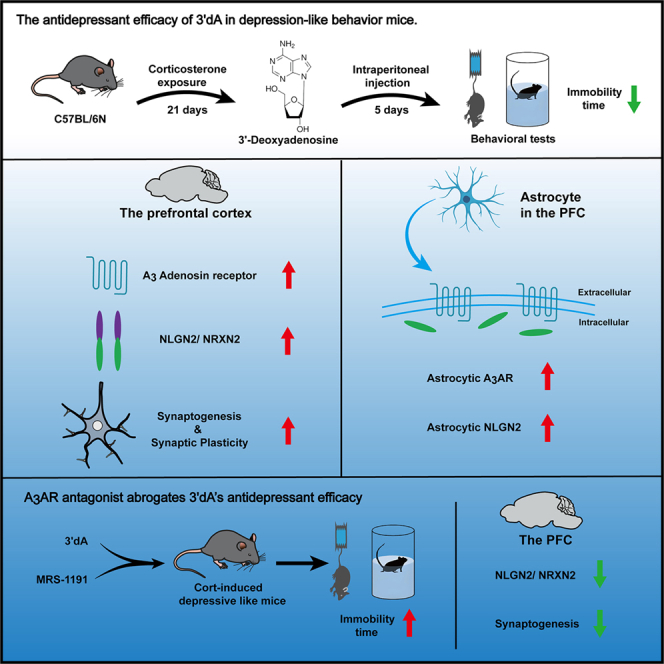

## Teaser

Molecular neuroscience; Neuroscience; Psychiatry

## Introduction

Understanding the molecular basis of major depressive disorder (MDD) remains one of the foremost challenges in modern medicine, with growing interest focused on synaptic dysfunction as a key pathophysiological mechanism.^1^ While the majority of studies in this field have investigated glutamatergic excitatory synapses in MDD, critical gaps persist in both mechanistic understanding and the development of safe, effective tools to modulate excitatory synaptic transmission.^2^ Elucidating these regulatory processes will not only advance our understanding of disease mechanisms underlying depression and related disorders but may also facilitate the identification of cell-specific therapeutic targets for precision interventions.

Research has shown that the prefrontal cortex (PFC) is robustly linked to the pathogenesis of depression.^3^ Neuroimaging evidence has revealed a significant reduction in PFC volume in depressed patients compared with that in healthy controls,^4^^,^^5^^,^^6^^,^^7^ and this volume loss can be effectively reversed by antidepressant treatment.^8^^,^^9^^,^^10^ Animal studies have further indicated that depressive-like behaviors in mouse models are associated not only with physiological and structural alterations in the amygdala and hippocampus (HIP), but also, and more importantly, with changes in the PFC.^11^^,^^12^ Astrocytes are the most abundant glial cells in the brain and play a multifaceted role in central nervous system (CNS) homeostasis by regulating neurotransmitter concentrations, facilitating neural and synaptic development, and promoting neuronal metabolism.^13^ Growing evidence now implicates dysfunctional astrocytes in the PFC as significant contributors to the pathophysiology of depression.^14^^,^^15^^,^^16^

A particularly compelling candidate molecule is the A_3_ adenosine receptor (A_3_AR), a member of the adenosine receptor family recently demonstrated to localize to microglia and astrocytes at the cellular level in the CNS, and A_3_AR has been shown to protect astrocytes from hypoxic damage.^17^^,^^18^^,^^19^ Nevertheless, the role of astrocytic regulation in modulating excitatory synapses in MDD remains poorly characterized. In the past 10 years, the list of known mechanisms of excitatory synapse organizers in depression has expanded dramatically due to major technological advances.^20^^,^^21^^,^^22^^,^^23^^,^^24^ A critical unresolved question is how these molecules were regulated specifically across excitatory synapses, or whether they modulate the excitatory synapse through glial-mediated mechanism.

3′-Deoxyadenosine (3′dA), an active compound found in the traditional Chinese medicinal herb *Cordyceps militaris*,^25^^,^^26^ exhibits a range of biological regulatory effects, including anti-tumor, anti-inflammatory, anti-thrombotic, anti-viral, anti-fungal, and hypoglycemic activities.^27^^,^^28^^,^^29^^,^^30^^,^^31^^,^^32^^,^^33^ Recent studies have revealed that 3′dA exerts neuroprotective effects by promoting synaptic plasticity and modulating neuroimmune responses within the CNS.^34^^,^^35^^,^^36^ Furthermore, literature suggests that the biological functions of 3′-dA are associated with the activation of A_3_AR.^37^^,^^38^^,^^39^^,^^40^^,^^41^ Molecular docking results indicate that 3′dA has a binding site on A_3_AR, which is consistent with that of adenosine.^42^ Additionally, 3′dA can upregulate the mRNA expression level of A_3_AR in cells.^43^

The neuroligin family of cell adhesion proteins—comprising Neuroligin-1, Neuroligin-2, and Neuroligin-3—is expressed in cortical glia (e.g., astrocytes and oligodendrocytes) and neurons, where it regulates neurogenesis through interactions with neuronal neurexins.^44^^,^^45^^,^^46^^,^^47^^,^^48^ Previous studies have demonstrated that abnormalities in neuroligin-2 (NLGN2) are associated with neuropsychiatric disorders, including autism spectrum disorder, anxiety, and intellectual disability.^49^^,^^50^^,^^51^ Notably, the absence of astrocytic NLGN2 has been shown to impair cortical excitatory synapse formation and function.^44^^,^^52^ Furthermore, downregulation of NLGN2 expression has been observed in depression mice, implicating its potential role in the synaptic pathophysiology of MDD.^53^ Recent research has revealed that the expressions of neuroligins in both glial cells (particularly astrocytes and oligodendrocytes) and neurons are high and involved in the regulation of synaptic function.^54^ Collectively, these findings highlight the critical roles of both neuronal and non-neuronal neuroligins in the CNS, underscoring their importance in synapse development and their potential as therapeutic targets for psychiatric disorders.

To date, however, the molecular, cellular, and circuit-level functions of astrocytic A_3_AR in the CNS, as well as its potential role in upregulating NLGN2 expression *in vivo*, remain unexplored. In this study, we investigated the contribution of A_3_AR to the antidepressant effects of 3′dA by addressing two central questions. First, do A_3_AR and/or NLGN2 act at specific excitatory synapses within depression-related circuitry, thereby offering synapse-specific targets for interventions? Second, is A_3_AR required for the 3′dA-induced antidepressant effect and the accompanying changes in excitatory synapse formation in this circuitry? Our findings implicate that A_3_AR plays a critical role in the regulation of astrocytic NLGN2 expression and excitatory synapse formation in the PFC. Moreover, these results raise the possibility that A_3_AR-expressing astrocytes contribute to the maintenance of excitatory synapse integrity in this region, highlighting them as potential targets for novel antidepressant therapies.

## Results

### Effect of 3′dA on depressive-like behaviors in corticosterone-treated mice

**Figure 1:**
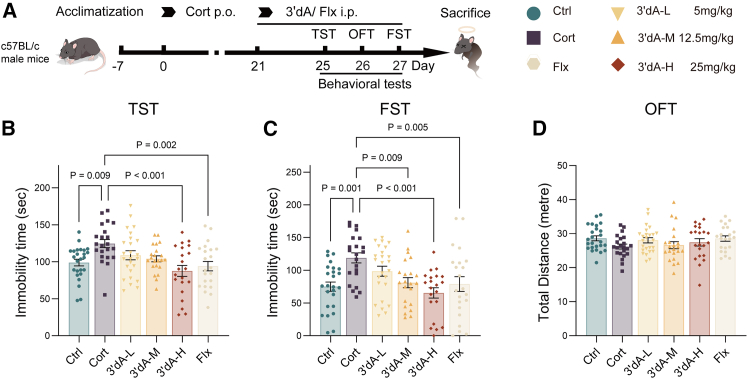
3′dA treatment reduces depressive-like behaviors in a corticosterone-induced model (A) Schematic of the experimental timeline for drug administration and behavioral assessments. (B and C) 3′dA significantly reduced immobility time in the tail suspension test (TST) and forced swim test (FST) (*n* = 21–25 mice/group). (D) 3′dA did not alter locomotor activity, as shown by the total distance traveled in the open-field test (OFT) (*n* = 21–25 mice/group). Data were analyzed by one-way ANOVA followed by Bonferroni post hoc test and are presented as mean ± SEM. *p* values are shown in the graphs.

The antidepressant effect of 3′dA was evaluated using a validated chronic corticosterone (Cort)-induced depression model in mice.^55^^,^^56^ Adult male C57BL/6N mice received corticosterone (25 μg/mL) dissolved in drinking water for 21 consecutive days to induce depressive-like behaviors. Subsequently, the mice were administered three different doses of 3′dA (5, 12.5, or 25 mg/kg) or fluoxetine (Flx, 15 mg/kg, positive control) daily for five days. Behavioral assessments were conducted using the tail suspension test (TST) and forced swim test (FST) (Figure 1A). Behavioral analyses revealed that chronic corticosterone administration significantly prolonged immobility durations in both the TST (Figure 1B; F (5, 131) = 4.861, *p* < 0.001) and FST (Figures 1C and 1F; F (5, 131) = 5.289, *p* < 0.001) compared with the control group, confirming the successful induction of depressive-like phenotypes. High-dose 3′dA (25 mg/kg) markedly reduced immobility times in the TST and FST relative to corticosterone-treated mice. Medium-dose 3′dA (12.5 mg/kg) significantly decreased immobility in the FST but showed no effect in the TST. In contrast, low-dose 3′dA (5 mg/kg) did not significantly alter immobility times in either test. We further determined whether 3′dA causes locomotor hyperactivity, using the open-field test (OFT) (Figure 1A). The data showed that 3′dA did not significantly affect the total distance traveled relative to controls (Figure 1D; F (5, 131) = 1.390, *p* = 0.232), suggesting that 3′dA did not induce locomotor hyperactivity in mice. Collectively, these results indicate that 3′dA administration produces a dose-dependent reduction in depressive-like behaviors in a chronic corticosterone-induced model, and the magnitude of this effect is comparable to that observed with the conventional antidepressant fluoxetine.

### Association between 3′dA treatment and A_3_AR expression in the PFC

**Figure 2:**
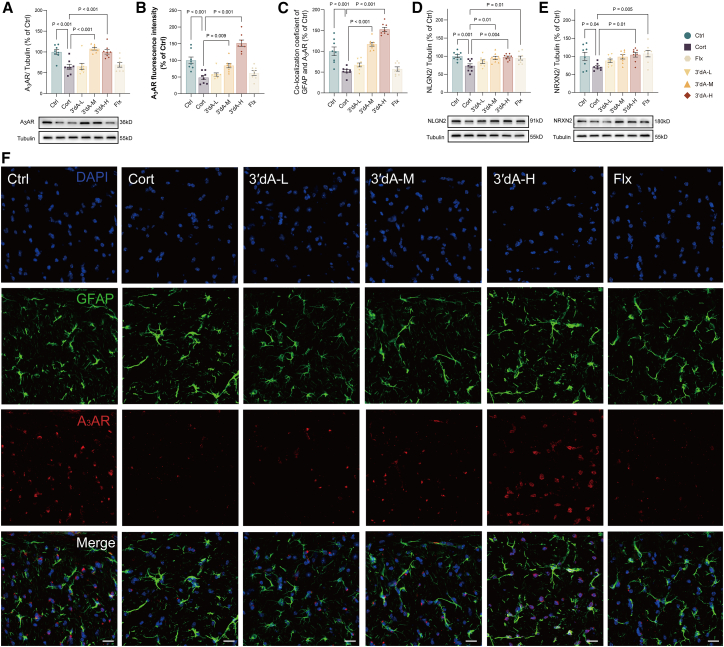
Expression of A_3_AR, NLGN2, and NRXN2 in the PFC following corticosterone and 3′dA treatments (A) Representative western blot and quantification of A_3_AR levels in the prefrontal cortex (PFC) (*n* = 8 mice/group). (B) Quantification of A_3_AR fluorescence intensity (*n* = 8 mice/group; 2 slices/mouse). (C) Co-localization coefficients for GFAP and A_3_AR (*n* = 8 mice/group; 2 slices/mouse). (D and E) Representative western blots and quantification of neuroligin-2 (NLGN2) and neurexin-2 (NRXN2) expression levels in the PFC (*n* = 8 mice/group). (F) Representative immunofluorescence images showing co-localization of astrocytes (GFAP, green) and A_3_AR (red) in the PFC. Scale bars, 20 μm. Data were analyzed by one-way ANOVA followed by Bonferroni post hoc test and are presented as mean ± SEM. *p* values are shown in the graphs.

To investigate whether chronic corticosterone exposure and 3′dA treatment are associated with changes in A_3_AR regulation, we quantified A_3_AR expression levels in the PFC of mice. Immunoblot analysis revealed that chronic corticosterone administration significantly downregulated A_3_AR expression in the PFC compared with the control group (Figure 2A; F (5, 42) = 12.58, *p* < 0.001). Notably, treatment with medium- and high-dose 3′dA significantly upregulated A_3_AR expression, restoring protein levels to near those of the control group. In contrast, neither low-dose 3′dA nor fluoxetine exerted a significant effect on A_3_AR expression. Consistent with these findings, immunofluorescence analysis further indicated that corticosterone treatment markedly reduced A_3_AR expression in the PFC (Figures 2B and 2F; F (5, 42) = 25.41, *p* < 0.001). Medium- and high-dose 3′dA not only increased A_3_AR expression but also enhanced its co-localization with glial fibrillary acidic protein (GFAP) (Figures 2C and 2F; F (5, 42) = 42.98, *p* < 0.001). However, neither low-dose 3′dA nor fluoxetine significantly altered A_3_AR expression or its co-localization with GFAP. In addition, GFAP-negative/A_3_AR-positive cells were observed in the 3′dA-treated groups, whereas such cells were absent in the “Cort” and “Flx” groups (Figure 2F). This suggests that 3′dA may promote A_3_AR expression not only in astrocytes but also in other cell types within the PFC.

### Changes in NLGN2 and neurexin-2 expression in the PFC following 3′dA treatment

Previous studies have demonstrated that NLGN2, a postsynaptic adhesion protein implicated in synaptic plasticity, plays a critical role in the pathophysiology and therapeutics of depression.^57^ To assess whether 3′dA modulates NLGN2 expression under chronic corticosterone exposure, we quantified NLGN2 expression levels in the PFC of mice. Our findings revealed that chronic corticosterone administration significantly reduced NLGN2 expression in the PFC compared with the control group (Figure 2D; F (5, 42) = 4.532, *p* = 0.002). Conversely, medium- and high-dose 3′dA treatments robustly upregulated NLGN2 expression, restoring it to near-control levels, whereas low-dose 3′dA elicited no significant changes. We further examined neurexin-2 (NRXN2), a primary presynaptic binding partner of NLGN2. Corticosterone-induced reductions in NRXN2 expression (Figures 2E and 2F; F (5, 42) = 3.419, *p* = 0.01) were effectively reversed by high-dose 3′dA in the PFC. Notably, the traditional antidepressant fluoxetine exhibited a similar regulatory effect on both NLGN2 and NRXN2 expression levels during this process.

### Association of 3′dA treatment with synaptic protein expression and spine density in the PFC

**Figure 3:**
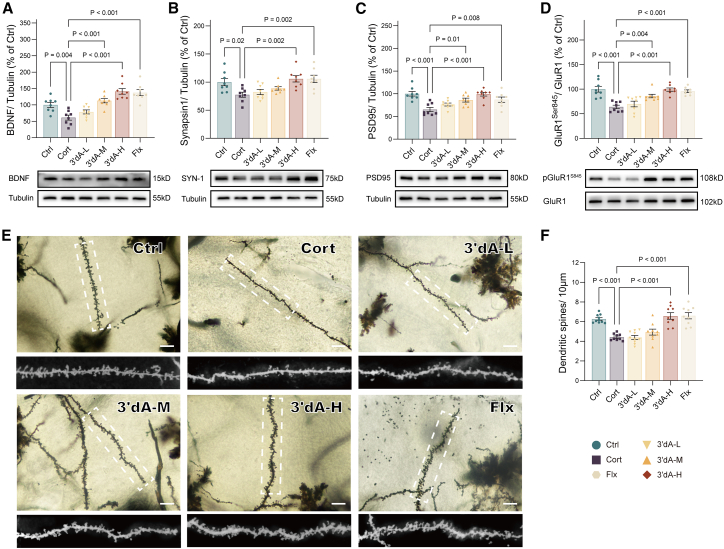
3′dA treatment is associated with increased synaptic protein expression and dendritic spine density in the PFC (A–D) Representative western blots and quantification of BDNF, synapsin-1, PSD-95, and GluR1 Ser845 levels in the PFC (*n* = 8 mice/group). (E and F) Representative Golgi-stained images and dendritic spine density in the PFC (*n* = 9–10 mice/group; 5 slices/mouse). Scale bars, 10 μm. Data were analyzed by one-way ANOVA followed by Bonferroni post hoc test and are presented as mean ± SEM. *p* values are shown in the graphs.

To explore synaptic changes associated with 3′dA treatment, we evaluated the expression of several proteins implicated in synaptogenesis and synaptic plasticity, processes that have been linked to antidepressant responses. Specifically, we measured the levels of brain-derived neurotrophic factor (BDNF), synapsin-1, PSD-95, and GluR1 phosphorylation at Ser845 in the PFC of corticosterone-treated mice following 3′dA administration. Immunoblot analysis revealed that chronic corticosterone exposure significantly downregulated the expression of BDNF (Figure 3A; F (5, 42) = 17.25, *p* < 0.001), synapsin-1 (Figure 3B; F (5, 42) = 5.430, *p* < 0.001), and PSD-95 (Figure 3C; F (5, 42) = 8.564, *p* < 0.001), as well as the phosphorylation of GluR1 at Ser845 (Figure 3D; F (5, 42) = 12.82, *p* < 0.001) in the PFC. In mice that were administered high-dose 3′dA for five consecutive days, the expression levels of these proteins and GluR1 phosphorylation at Ser845 were comparable to those observed in control mice. Medium-dose 3′dA produced variable effects across markers, whereas low-dose 3′dA had no significant impact. These findings are consistent with the behavioral outcomes. Notably, fluoxetine-treated mice also exhibited BDNF, synapsin-1, PSD-95, and GluR1 phosphorylation levels that did not differ significantly from controls, a pattern similar to that seen with high-dose 3′dA treatment.

To further explore the effects of 3′dA on synaptic morphology, we assessed dendritic spine density in the PFC, using Golgi staining. The results demonstrated that both high-dose 3′dA and fluoxetine significantly increased the dendritic spine density (Figures 3E and 3F; F (5, 50) = 18.99, *p* < 0.001) in corticosterone-exposed mice, whereas medium- and low-dose 3′dA had no significant effect.

### Differential association of 3′dA and fluoxetine with astrocytic NLGN2 and the NLGN2-NRXN2 complex

**Figure 4:**
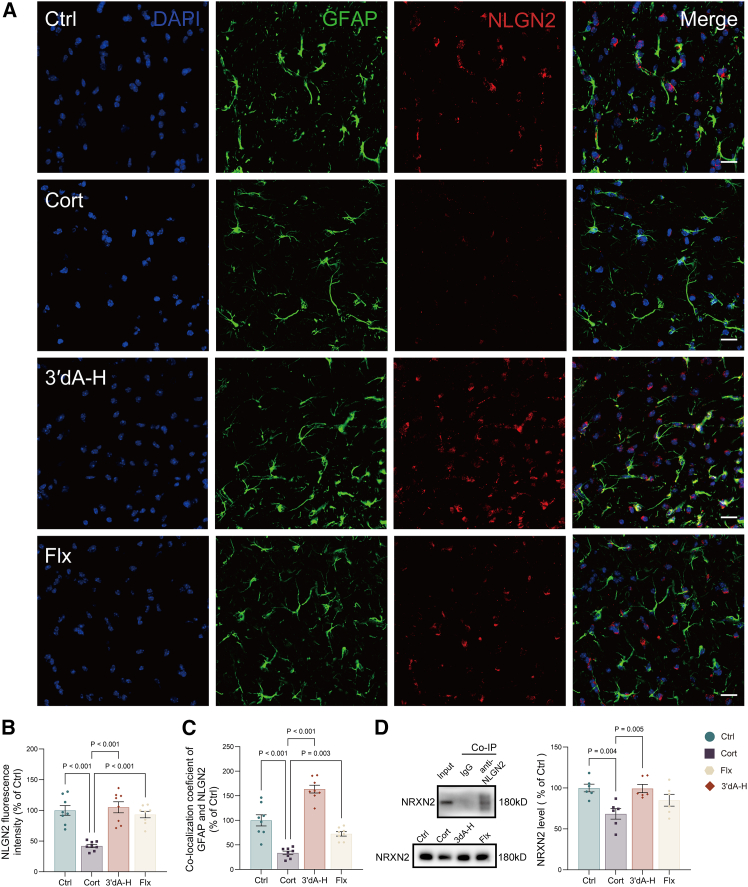
3′dA treatment is associated with enhanced astrocytic NLGN2 co-localization and NLGN2-NRXN2 complex formation (A) Representative immunofluorescence images showing co-localization of astrocytes (GFAP, green) and NLGN2 (red) in the PFC. Scale bars, 20 μm. (B) Quantification of NLGN2 fluorescence intensity (*n* = 8 mice/group; 4 slices/mouse). (C) Co-localization coefficients for GFAP and NLGN2 (*n* = 8 mice/group; 4 slices/mouse). (D) Representative co-immunoprecipitation blot and quantification of NLGN2-NRXN2 complex (*n* = 6 mice/group). Data were analyzed by one-way ANOVA followed by Bonferroni post hoc test and are presented as mean ± SEM. *p* values are shown in the graphs.

Emerging evidence underscores the critical role of astrocytic NLGN2 and its interaction with NRXN2 in regulating synaptogenesis and synaptic plasticity.^58^ In this study, we determined whether 3′dA treatment is associated with changes in astrocytic NLGN2 expression and NLGN2-NRXN2 complex formation. Immunofluorescence staining revealed that chronic corticosterone exposure significantly reduced NLGN2 expression in the PFC compared with the control group (Figures 4A and 4B; F (3, 28) = 18.87, *p* < 0.001). Both high-dose 3′dA and fluoxetine restored NLGN2 levels, consistent with western blot findings. However, 3′dA—but not fluoxetine—significantly enhanced NLGN2 co-localization with GFAP in the PFC (Figures 4A and 4C; F (3, 28) = 53.42, *p* < 0.001), suggesting a unique mechanism by which 3′dA promotes astrocytic NLGN2 expression to mediate its antidepressant effects. Furthermore, co-immunoprecipitation analysis demonstrated that the corticosterone-induced disruption of the NLGN2-NRXN2 complex (Figure 4D; F (3, 20) = 6.253, *p* = 0.004) was selectively reversed by high-dose 3′dA, whereas fluoxetine exhibited no significant effect. These data suggest that 3′dA, but not fluoxetine, selectively restored astrocytic NLGN2 and NLGN2-NRXN2 complex levels under conditions of chronic corticosterone exposure.

### The A_3_AR antagonist MRS-1191 blocks the behavioral and molecular effects of 3′dA

**Figure 5:**
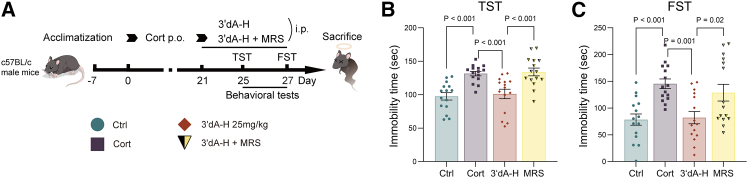
The A_3_AR antagonist MRS-1191 abolishes the behavioral effects of 3′dA (A) Schematic of the experimental timeline for drug treatment and behavioral tests. (B and C) MRS-1191 abolished the reduction in immobility time induced by 3′dA in TST and FST (*n* = 15 mice/group). Data were analyzed by one-way ANOVA followed by Bonferroni post hoc test and are presented as mean ± SEM. *p* values are shown in the graphs.

**Figure 6:**
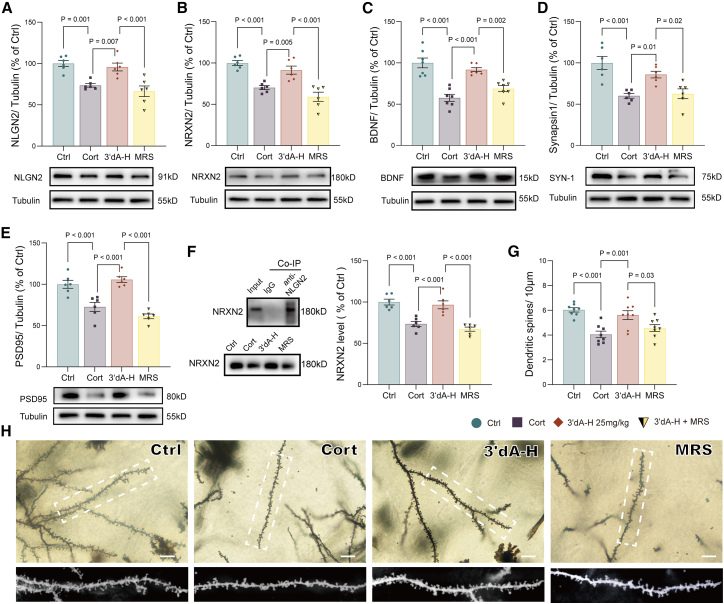
MRS-1191 blocks the effects of 3′dA on molecular and synaptic markers (A–E) Representative western blots and quantification of NLGN2, NRXN2, BDNF, synapsin-1, and PSD-95 levels in the PFC (*n* = 6–7 mice/group). (F) Representative co-immunoprecipitation blot and quantification of NLGN2-NRXN2 complex levels (*n* = 6 mice/group). (G and H) Representative Golgi-stained images and dendritic spine density in the PFC (*n* = 8 mice/group; 5 slices/mouse). Scale bars, 10 μm. Data were analyzed by one-way ANOVA followed by Bonferroni post hoc test and are presented as mean ± SEM. *p* values are shown in the graphs.

To test the hypothesis that the antidepressant effects of 3′dA depend on A_3_AR activation, we examined whether the selective A_3_AR antagonist MRS-1191 could block these effects in the chronic corticosterone-induced depression model. The mice were pretreated with MRS-1191 (1 mg/kg, i.p.) prior to 3′dA administration (25 mg/kg, i.p.) for five consecutive days (Figure 5A). Behavioral assessments via TST and FST revealed that MRS-1191 pretreatment abolished 3′dA-induced reductions in immobility times (TST: Figure 5B; F (3, 56) = 12.07, *p* < 0.001; FST: Figure 5C; F (3, 56) = 7.909, *p* < 0.001). At the molecular level, immunoblot analysis revealed that MRS-1191 attenuated 3′dA-mediated upregulation of NLGN2 (Figure 6A; F (3, 20) = 13.44, *p* < 0.001), NRXN2 (Figure 6B; F (3, 20) = 20.72, *p* < 0.001), BDNF (Figure 6C; F (3, 24) = 21.46, *p* < 0.001), synapsin-1 (Figure 6D; F(3, 20) = 11.82, *p* < 0.001), and PSD-95 (Figure 6E; F(3, 20) = 25.12, *p* < 0.001) in the PFC. Co-immunoprecipitation assays further demonstrated that MRS-1191 suppressed 3’dA’s ability to restore the NLGN2-NRXN2 complex (Figure 6F; F (3, 20) = 20.94, *p* < 0.001) in the PFC. Morphologically, MRS-1191 negated 3’dA’s enhancement of dendritic spine density (Figures 6G and 6H; F (3, 28) = 11.30, *p* < 0.001) in the PFC. Taken together, these findings suggest that the behavioral and molecular changes associated with 3′dA treatment are prevented by pharmacological blockade of A_3_AR.

## Discussion

In this study, we investigated the potential role of A_3_AR in regulating excitatory synapse formation in prefrontal cortical circuitry following 3′dA administration. Our findings indicate that the administration of the adenosine receptor activator 3′dA produces robust and sustained antidepressant effects in chronic corticosterone-exposed mice and that these effects are not accompanied by locomotor hyperactivity, which distinguishes 3′dA’s effects from those of psychostimulant antidepressants.^59^^,^^60^ Furthermore, 3′dA treatment was associated with the restoration of A_3_AR levels and increased expression of NLGN2 and NRXN2, as well as enhanced NLGN2-NRXN2 complex formation in the PFC of corticosterone-exposed mice. These molecular changes were accompanied by increases in neuroplasticity-related signaling markers, including BDNF, synapsin-1, PSD-95, and GluR1 phosphorylation at Ser845, as well as higher dendritic spine density in the PFC. Notably, increased NLGN2 immunoreactivity was observed to co-localize with astrocytes in the 3′dA-treated group, whereas this pattern was not detected in fluoxetine-treated mice, suggesting that 3′dA treatment may influence astrocytic NLGN2 expression. Pharmacological blockade of A_3_AR with MRS-1191 prevented the 3′dA-associated reductions in immobility time and the accompanying molecular changes, including the increases in NLGN2, NRXN2, BDNF, and spine density, as well as the restoration of NLGN2-NRXN2 complex levels. These results suggest that A_3_AR activation is necessary for the behavioral and molecular changes observed with 3′dA treatment.

3′dA is an adenosine receptor activator, and modulation of the A_3_AR by this compound plays an important role in its anti-cancer mechanisms.^27^ In addition, activation of A_3_AR by Cl-IBMECA (an A_3_AR specific agonist) increased the magnitude of theta-burst-induced long-term potentiation (LTP) and attenuated long-term depression (LTD), suggesting a role of A_3_AR in regulation of the excitatory synaptic plasticity.^61^ Moreover, A_3_AR plays a critical role in the regulation of ischemic diseases as well as in inflammatory and autoimmune pathologies.^62^ In rodents, A_3_AR is expressed mainly in the testes, lung, kidneys, heart, and brain.^63^ The first second-messenger systems found to be associated with A_3_AR activation were adenylyl cyclase (AC) activity, which is inhibited, and phospholipase C (PLC), which is stimulated, through Gi and Gq protein coupling, respectively.^64^ Activation of A_3_AR is responsible for mitochondrial intracellular calcium (Ca^2+^) modulation, which is also implicated in depression.^65^ Our data suggest that A_3_AR may be an important modulator of NLGN2 expression and excitatory synapse formation in the PFC and that A_3_AR-expressing astrocytes could contribute to the support of excitatory synapses, thereby representing a potential target for antidepressant therapies (Figures 2, 4, 5, and 6). We propose that the modulation of NLGN2 levels in the PFC, potentially through the regulation of astrocyte function, may facilitate adaptive behavioral responses to stress.

BDNF (a neuropeptide) serves as a key regulator in the pathogenesis of depression.^66^^,^^67^ It is primarily expressed in glutamatergic neurons and astrocytes in both PFC and HIP,^68^^,^^69^ where it modulates synaptogenesis and synaptic plasticity.^70^^,^^71^ Studies have demonstrated that BDNF deficiency leads to suppressed dendritic growth,^72^ whereas BDNF stimulation promotes dendritic outgrowth and spine formation in primary hippocampal cultures grown in B27-depleted medium.^73^ Acute application of BDNF results in dendritic spine head size augmentation, spine length elongation, and increased filopodia protrusions.^74^ Chronic BDNF treatment enhances apical dendritic length in hippocampal slice cultures,^75^ and the overexpression of BDNF in hippocampal neurons induces maturation of both excitatory and inhibitory synapses.^76^ Furthermore, BDNF facilitates LTP through activation of the tropomycin receptor kinase B (TrkB) receptor.^69^ Our results demonstrated that 3′dA effectively increased the expression levels of BDNF, synapsin-1, PSD-95, and pGluR1^S845^, as well as dendritic spine density, in the PFC of a mouse model of corticosterone-induced depressive-like behavior. (Figure 3), and the regulatory effects of 3′dA on BDNF, synapsin-1, PSD-95, and dendritic spine density were effectively suppressed by a selective A_3_AR antagonist (Figure 6).

NLGN2—a postsynaptic adhesion protein—plays a critical role in the pathophysiology of depression by mediating synaptogenesis and synaptic plasticity through its interaction with presynaptic NRXN2.^54^ Furthermore, astrocytic NLGN2 regulates the excitatory/inhibitory synaptic balance, and emerging evidence suggests that synaptic pathologies linked to neuroligin mutations may arise from dysregulated astrocyte-mediated synaptic modulation.^77^ A recent study showed that glial progenitor cells from patients with schizophrenia express significantly lower levels of NLGN-1, NLGN2, and NLGN-3 than those from controls.^78^ NLGN-3 and NLGN-4 mutants are strongly implicated as candidates molecules underlying the development of neuropsychiatric disorders with social disturbances such as autism.^79^ However, NLGN2 gene expression in the nucleus accumbens (NAc) of patients with MDD was significantly reduced.^53^ In the absence of astrocytic NLGN2, the formation of cortical excitatory synapses is diminished but inhibitory synaptic function is enhanced.^52^ We observed reduced NLGN2 levels in the PFC of depressive-like mice (Figure 2); these levels were restored by 3′dA treatment. Thus, modulation of NLGN2 may represent a correlate of antidepressant behavioral changes. We also detected reduced NLGN2-NRXN2 complex levels in the PFC of depressive-like mice (Figure 2). The restoration of this complex by 3′dA treatment was paralleled by increases in excitatory synaptic markers (synapsin-1, PSD-95, and GluR1), BDNF expression, and dendritic spine density in the PFC (Figures 2 and 3). Notably, the enhanced NLGN2 immunoreactivity co-localized with the astrocytic marker GFAP in the 3′dA-treated group, raising the possibility that this effect may involve astrocytic A_3_AR (Figure 4). In contrast, fluoxetine treatment increased the total NLGN2 levels but did not detectably enhance astrocytic NLGN2 co-localization, suggesting that fluoxetine may act through distinct mechanisms (Figure 4). Although both 3′dA and fluoxetine increased the total NLGN2 levels in the PFC, only 3′dA was associated with elevated astrocytic NLGN2. This difference may reflect distinct upstream signaling pathways, while both treatments converged on enhanced synaptogenesis as a common downstream outcome. Furthermore, pretreatment with the A_3_AR antagonist MRS-1191 prevented the 3′dA-associated increase in NLGN2-NRXN2 complex levels, BDNF expression, and dendritic spine density (Figures 5 and 6). Taken together, these data suggest that A_3_AR and NLGN2 may be important mediators of the antidepressant effects of 3′dA.

**Figure 7:**
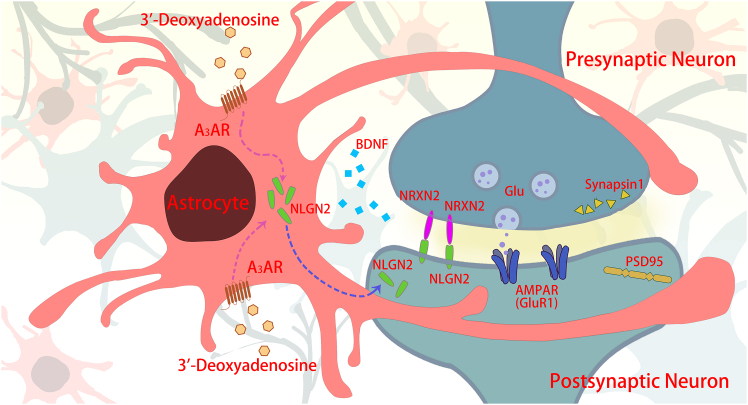
Proposed model of 3′dA action involving astrocytic A_3_AR and synaptic remodeling

In summary, our findings indicate that 3′dA treatment is associated with increased NLGN2 expression in astrocytes, an effect that may involve A_3_AR activation and may contribute to its antidepressant effects. Pharmacological blockade of A_3_AR abolished the antidepressant behavioral response and prevented the restoration of excitatory synapse formation. Collectively, these results raise the possibility that A_3_AR signaling may facilitate excitatory synapse formation in the PFC, in part through the upregulation of NLGN2 expression, a process that could be relevant for the circuitry underlying antidepressant-like behaviors (Figure 7). The present study, by demonstrating that 3′dA treatment modulates astrocytic NLGN2 expression via A_3_AR, provides new insights into the psychopharmacology of depression and may inform the future development of therapeutic strategies aimed at promoting resilience mechanisms for the treatment of depression.

### Limitations of the study

Although accumulating research has identified several brain regions—including the PFC, HIP, NAc, and basolateral amygdala (BLA)—as playing important roles in stress responses,^80^^,^^81^^,^^82^ the PFC serves as a key hub in the brain for regulating emotions, social behavior, cognitive processes, and executive functions.^3^^,^^83^^,^^84^^,^^85^ Multiple longitudinal studies have shown that in untreated patients with recurrent depression, the gray matter volume of the PFC—especially the subgenual anterior cingulate cortex and dorsolateral regions—is markedly reduced.^86^^,^^87^^,^^88^ This region is a key site for the action of glutamate and monoamine neurotransmitters, such as serotonin and dopamine. Its central role explains why classic antidepressants (e.g., selective serotonin reuptake inhibitors [SSRIs]) work by increasing serotonin levels in this region,^89^ and why the left dorsolateral PFC is currently the standard target for clinically effective repetitive transcranial magnetic stimulation (rTMS).^90^ Simply put, the neuroscientific essence of depression lies in a failure of the PFC’s “top-down” regulation of emotional centers. In future studies, electrophysiological investigations using patch-clamp recordings and electroencephalography (EEG) are warranted to examine the modulatory effects of 3′dA on brain electrophysiological signals in mice exhibiting corticosterone-induced depression-like behaviors. These experiments will provide further evidence that the activation of A_3_AR in the PFC is key to the antidepressant effect of 3′dA. Therefore, further studies are warranted to downregulate A_3_AR in astrocytes and NLGN2 in prefrontal cortical neurons, which would help elucidate whether A_3_AR-mediated signaling in the PFC represents a key mechanism driving this antidepressant effect.

## Resource availability

### Lead contact

Requests for further information, resources, and reagents should be directed to and will be fulfilled by the lead contact, Bai Li (libai@ynu.edu.cn).

### Materials availability

This study does not involve any new unique materials.

### Data and code availability

• All data supporting the findings of this study are available from the lead contact upon reasonable request.
• This study does not report any original code.
• Any additional information required to reanalyze the data reported in this paper is available from the lead contact upon request.

## Acknowledgments

The authors express their sincere gratitude to Ms. Kajia Zhang for her invaluable assistance in data analysis and figure preparation. This work was supported by the 10.13039/501100001809National Natural Science Foundation of China (grant no. 81960657), the Science and Technology Innovation Fund of China “Brain Science and Brain-Like Research” (grant no. 2021ZD0202003), the 10.13039/501100018531Science and Technology Project of Yunnan Province (grant no. 202201AT070298), and Yunnan Provincial Key Research and Development Plan (grant no. 202403AC100020).

## Author contributions

Conceptualization, B.L., C.X., and J.D.; formal analysis, M.Z., W.H., and D.P.; investigation, M.Z., J.L., W.H., and D.P.; methodology, M.Z. and J.L.; validation, B.L.; visualization, B.L.; writing – original draft, B.L.; writing – review & editing, B.L. and J.D.; funding acquisition, B.L., C.X., and J.D.; project administration, B.L.; supervision, J.D.

## Declaration of interests

The authors declare no competing interests.

## STAR★Methods

### Key resources table

**Table:** 

REAGENT or RESOURCE	SOURCE	IDENTIFIER
**Antibodies**		

A_3_AR antibody	Biorbyt	Cat#orb393197, RRID:AB_3751512
NLGN2 antibody	Synaptic Systems	Cat#129203, RRID:AB_993014
NRXN2 antibody	abcam	Cat#ab34245, RRID:AB_776702
Synapsin-1 antibody	abcam	Cat#ab254349, RRID:AB_2920663
PSD-95 antibody	abcam	Cat#ab12093, RRID:AB_298846
pGluR1 Ser845 antibody	abcam	Cat#ab76321, RRID:AB_1523688
BDNF antibody	Proteintech	Cat#28205-1-AP, RRID:AB_2818984
GluR1 antibody	Affinity Biosciences	Cat#AF6306, RRID:AB_2835154
GFAP antibody	Affinity Biosciences	Cat#BF8023, RRID:AB_3751513
Tubulin antibody	Affinity Biosciences	Cat#T0023, RRID:AB_2813772
Goat anti-Rabbit IgG (H + L) Secondary Antibody, HRP	Thermo Fisher Scientific	Cat#31460, RRID:AB_228341
Goat anti-Mouse IgG (H + L) Secondary Antibody, HRP	Thermo Fisher Scientific	Cat#31430, RRID:AB_228307
Alexa Fluor 488-AffiniPure Goat Anti-Mouse IgG (H + L)	Jackson ImmunoResearch	Cat#115-545-003, RRID:AB_2338840
Alexa Fluor 594 AffiniPure Donkey Anti-Rabbit IgG (H + L)	Jackson ImmunoResearch	Cat#711-585-152, RRID:AB_2340621
Rabbit IgG	Beyotime	Cat#A7016, RRID:AB_2905533

**Biological samples**		

mice brain	This paper	Not applicable

**Chemicals, peptides, and recombinant proteins**		

3′-Deoxyadenosine	Yuanye	Cat#B20196
Corticosterone	TCI	Cat#C0388
fluoxetine	TCI	Cat#F0750
MRS-1191	MCE	Cat#HY-124543
protease inhibitors	Roche	Cat#04693132001
phosphatase inhibitors	Roche	Cat#04906845001
bovine serum albumin	Sangon Biotech	Cat#A600332
isoflurane	RWD	Cat#R510-22
radioimmunoprecipitation assay	Beyotime	Cat#P0013K
lysis buffer	Beyotime	Cat#P0013J
DAPI-containing antifade mounting medium	Beyotime	Cat#P0131
glycerol jelly mounting medium	Beyotime	Cat#C0187
DMSO	Solarbio	Cat#ID9011
Tween-80	Solarbio	Cat#IR9096
Tween 20	Solarbio	Cat#IT9010
phosphate-buffered saline	Solarbio	Cat#P1020
paraformaldehyde	Solarbio	Cat#P1110
Triton X-100	Solarbio	Cat#T8200
protein loading buffer	Takara Bio	Cat#9173

**Critical commercial assays**		

bicinchoninic acid assay kit	Beyotime	Cat#P0012
Golgi-Cox staining kit	FD Neurotechnologies	Cat#PK401

**Experimental models: Organisms/strains**		

adult male C57BL/6N mice	Charles River	https://www.vitalriver.com/

**Software and algorithms**		

SMART software (v3.0)	Panlab	https://www.panlab.com/en/
ImageJ software (1.54m)	The National Institutes of Health	https://www.imagej.net/
GraphPad Prism (v10.2.2)	GraphPad Software	https://www.graphpad.com/
Adobe Illustrator (v26.0.3)	Adobe	https://www.adobe.com/

**Other**		

PVDF Membrane	Millipore	Cat#IPVH00010
magnetic protein A/G beads	Thermo Fisher Scientific	Cat#88802

### Experimental model and study participant details

#### Animals

Adult male C57BL/6N mice (7 weeks old, 20–22 g) were group-housed (4 per cage) under a 12-h light-dark cycle at a constant temperature (22 ± 2°C) and humidity (55% ± 5%), with *ad libitum* access to food and water. Following a 1-week acclimatization period, the mice were randomly assigned to different treatment groups prior to the commencement of the experiments.

The chronic corticosterone-induced depressive-like behavior model, which elevates glucocorticoid levels, is a well-established model for studying depression.^55^^,^^56^^,^^91^ Corticosterone (Cort, 25 μg/mL) was dissolved in a vehicle solution (0.05% DMSO and 0.05% Tween-80 in water) and stored in opaque bottles to prevent light exposure. For 21 days, the drinking water of the corticosterone-treated groups was replaced with the corticosterone solution, while the control group received the vehicle alone. After the 3-week treatment period, the corticosterone-treated mice were randomly divided into five groups and received the following via intraperitoneal (i.p.) injection (10 μL/g): (1) saline, (2) low-dose 3′-deoxyadenosine (3′dA, 5 mg/kg), (3) medium-dose 3′dA (12.5 mg/kg), (4) high-dose 3′dA (25 mg/kg), or (5) fluoxetine (Flx, 15 mg/kg). The vehicle-treated control group received saline injections. Following 5 consecutive days of drug or vehicle administration, the mice were subjected to the tail suspension test (TST) on day 25, the open field test (OFT) on day 26, and the forced swimming test (FST) on day 27.

In a separate experiment, another cohort of mice was treated with corticosterone for 21 days under similar conditions and randomly assigned to three groups: (1) Cort + saline, (2) Cort + high-dose 3′dA (25 mg/kg), and (3) Cort + high-dose 3′dA + MRS-1191 (MRS, a selective A3 adenosine receptor antagonist, 1 mg/kg). The control group was treated as described above. After 5 consecutive days of drug or vehicle administration, the mice were subjected to the TST and FST under the same experimental settings.

#### Ethics statement

All animal care, treatments, and experimental procedures were conducted in compliance with the *Guide for the Care and Use of Laboratory Animals* and were approved by the Laboratory Animal Welfare Ethics Committee of Yunnan University (approval number: YNU20210118).

### Method details

#### Behavioral tests

##### Tail suspension test (TST)

Each mouse was individually suspended upside down by its tail from a bar positioned 30 cm above the ground. The behavior was recorded for 6 min, and the immobility time during the final 4 min was quantified using SMART software.

##### Open field test (OFT)

An activity chamber (40 × 40 × 20 cm) with a white floor virtually divided into 16 equal squares. Each mouse was individually placed in the center of the chamber and video-recorded for 6 min. The total distance traveled was automatically analyzed using SMART software. The chamber was cleaned during the interval of test.

##### Forced swimming test (FST)

Each mouse was placed individually in a cylindrical container (height: 40 cm, diameter: 20 cm) filled with 25 cm of water (22 ± 1°C) and forced to swim for 6 min. The session was recorded, and the duration of immobility during the last 4 min was analyzed by a blinded observer. Immobility was defined as the absence of struggling, making only necessary movements to keep its head above the water.

#### Western blot

Mice were euthanized by cervical dislocation. Prefrontal cortex tissues were dissected from the brain, immediately frozen in liquid nitrogen, and homogenized in radioimmunoprecipitation assay (RIPA) buffer containing protease and phosphatase inhibitors at 4°C. Protein concentrations were determined using a bicinchoninic acid (BCA) assay kit. Denatured protein samples (10 μg/well) were separated on 7–12% SDS-polyacrylamide gels and transferred to polyvinylidene difluoride (PVDF) membranes. The membranes were blocked with 1% bovine serum albumin for 1 h at room temperature and then incubated overnight at 4°C with the following primary antibodies: A_3_AR (1:300), NLGN2 (1:1000), NRXN2 (1:1000), BDNF (1:1000), Synapsin-1 (1:1000), PSD-95 (1:1000), GluR1 (1:1000), pGluR1 Ser845 (1:1000), or Tubulin (1:5000). After washing with TBST (0.1% Tween 20), the membranes were incubated for 1 h at room temperature with horseradish peroxidase-conjugated secondary antibodies: goat anti-rabbit (1:10,000) or goat anti-mouse (1:5,000). Immunoreactive bands were visualized using an enhanced chemiluminescent system (Imager 600, Amersham) and quantified using ImageJ software.

#### Immunofluorescence

Mice were deeply anesthetized with isoflurane and perfused with phosphate-buffered saline (PBS) followed by 4% paraformaldehyde (PFA) in PBS. Brain tissues were fixed in 4% PFA for 12 h and then immersed in a gradient sucrose solution (10%, 20%, 30% w/v) for 3 days at 4°C. Cryosections (10 μm thick) were washed in PBST (PBS with 0.3% Triton X-100), blocked with 3% bovine serum albumin for 1 h at room temperature, and incubated overnight at 4°C with primary antibodies: GFAP (1:200, BF8023), A_3_AR (1:100) and NLGN2 (1:200). The sections were then washed in PBST and incubated for 1 h at room temperature with Alexa Fluor-conjugated secondary antibodies: goat anti-mouse 488 (1:500) and donkey anti-rabbit 594 (1:500). The sections were mounted with DAPI-containing antifade mounting medium. Images were acquired using a confocal laser scanning microscope (FV1000, Olympus) and analyzed using ImageJ software.

#### Golgi-cox staining

Dendritic spine density was assessed using a commercial Golgi-Cox staining kit. Briefly, mice were anesthetized with isoflurane, perfused with PBS, and fixed with 4% PFA in PBS. Brains were rinsed in distilled water to remove surface blood and immersed in an impregnation solution (equal volumes of solutions A and B) for 2 weeks in the dark at room temperature. The brains were then transferred to solution C and stored for 1 week in the dark at room temperature. Brain sections (100 μm thick) were cut using a cryostat, stained in a solution (1 part solution D, 1 part solution E, and 2 parts distilled water) for 10 min, washed, dehydrated in a graded ethanol series (50%, 75%, 95%, and 100%), and cleared in xylene. The sections were mounted with glycerol jelly mounting medium and imaged using an optical microscope (Axio Imager A2, ZEISS). Dendritic spine density was quantified using ImageJ software.

#### Co-immunoprecipitation

Prefrontal cortex tissues were homogenized in lysis buffer containing protease and phosphatase inhibitors for 30 min at 4°C. The homogenates were centrifuged at 12,000 × g for 10 min, and the protein concentration of the supernatant was determined using a BCA assay kit. The samples were incubated overnight at 4°C with NLGN2 antibody (1:100) or normal rabbit IgG. Magnetic protein A/G beads were added to the mixture and incubated for 4 h at 4°C. The beads were collected using a magnetic stand, washed in lysis buffer, and the immunocomplexes were eluted in protein loading buffer. The eluted proteins were subjected to Western blot analysis as described above. Protein samples (50 μg/well) were separated on 12% SDS-polyacrylamide gels, transferred to PVDF membranes, and blocked with 1% bovine serum albumin for 1 h at room temperature. The membranes were incubated with NRXN2 antibody (1:1000) overnight at 4°C, washed in TBST (0.1% Tween 20), and incubated with horseradish peroxidase-conjugated goat anti-rabbit secondary antibody (1:10,000) for 1 h at room temperature. Immunoreactive bands were visualized using an enhanced chemiluminescent system (Imager 600, Amersham) and quantified using ImageJ software.

### Quantification and statistical analysis

All data were analyzed using one-way ANOVA followed by Bonferroni post hoc tests. Results are presented as mean ± SEM. A *p*-value <0.05 was considered statistically significant. Figures were generated using GraphPad Prism and Adobe Illustrator.
